# Inferring Mental States from Brain Data: Ethico‐legal Questions about Social Uses of Brain Data

**DOI:** 10.1002/hast.4958

**Published:** 2025-03-05

**Authors:** Jennifer A. Chandler

**Keywords:** neuroethics, brain‐computer technologies, mental privacy, brain‐data privacy, human rights, neurorights, neurotechnology

## Abstract

*Neurotechnologies that collect and interpret data about brain activity are already in use for medical and nonmedical applications. Refinements of existing noninvasive techniques and the discovery of new ones will likely encourage broader uptake. The increased collection and use of brain data and, in particular, their use to infer the existence of mental states have led to questions about whether mental privacy may be threatened. It may be threatened if the brain data actually support inferences about the mind or if decisions are made about a person in the belief that the inferences are justified. This article considers the chain of inferences lying between data about neural activity and a particular mental state as well as the ethico‐legal issues raised by making these inferences, focusing here on what the threshold of reliability should be for using brain data to infer mental states*.

## Neuroscience & Society

*How should we think about technologies that seem to read the mind? The first of a three‐part series.*

A range of emerging neurotechnologies aims to “read from” the brain, by collecting and interpreting data about brain structure and activity, and “write to” the brain, by modifying brain activity through electromagnetic, optical, sonic, or other techniques. These technologies are therapeutically promising. Some are already licensed for some medical uses,^1^ and new uses are being actively researched.^2^ The technologies also raise concerns that have generated widespread scholarly and increasingly public debate.

What might be the consequences of inferring mental states from data about brain structure and activity—“brain data,” for simplicity's sake—and how does the capability of making such inferences affect personal privacy? What ethical and legal guardrails should be in place to protect research participants and device users? These neurotechnologies will almost certainly not be limited to medical applications that are controlled through existing medical regulatory systems and professional ethical codes. In all likelihood, their use will expand into a broad range of wellness, entertainment, employment, legal, correctional, military, marketing, and other social domains.^3^ To strengthen the guardrails, a set of novel human rights intended to address concerns about the potential uses of these emerging neurotechnologies—“neurorights”—have been proposed to address unwanted consequences at both individual and societal levels.^4^

Consider, for example, a proposed right to mental privacy.^5^ The idea that the mental sphere is personal and private is implicit in long‐standing legal protections related to the privacy of personal information and private spaces.^6^ To the extent that others can access a person's mind, that person's mental privacy could be imperiled. We have long drawn inferences about other people's mental states just from observing and interpreting their behavior, and some physiological data (such as sweating and heart rate) has also been available to us. The access to mental states that such observations afford is familiar, of course, and is part of the reason that private spaces are legally protected—to offer a refuge from the eyes of others and the inferences they might draw about us from their observations.

What does brain data add, and how could the collection and use of that data threaten mental privacy? On their surface, brain data, as defined here, merely reveal the structure or fluctuating hemodynamic, metabolic, or electrical properties of the brain. These physiological data are personal bodily information, and we might feel that it is private for the same reasons that other types of physiological information, such as heart rate, blood pressure, and blood glucose levels, are considered private. But none of the brain data directly reveal mental states; to threaten mental privacy, the data must allow one to draw an inference from them to a mental state.

The purpose of this paper is to consider the ethical and legal implications of drawing such inferences and then of applying those conclusions in a range of cases, both actual and potential. The paper will first provide a high‐level sketch of the inferential steps between brain data and mental states. As will be seen, there is a complex chain linking brain data to mental states, made up of multiple possible technologies “reading out” from the brain and multiple possible contexts in which the inferred mental states will be of interest. The ethical evaluation of these inferences is similarly complex, with a chain of inferential steps from brain data to mental states and often an additional chain from a mental state—like memory—to the information of ultimate interest—a past event. The inferential chain described here is meant to be generic—applicable across a range of types of brain data and a range of techniques for detection, analysis, and decoding of data—and the main intended contribution is to explore the ethics of following this inferential chain within broader sociolegal contexts in which the inferences might be drawn.

Mental privacy is not the only value at stake in making these inferences. Autonomy, dignity, and mental and physical integrity are also at stake and might in fact require trade‐offs against privacy. For example, a person who cannot communicate because of a severe mobility impairment would benefit from a device that could decode imagined speech from brain activity. A person's perception or memory of an event could be critically important in a legal matter. Distinguishing true and false memories (an ability we do not yet have) might help protect against the risk of accepting confabulated false confessions.

## Clarifying Terms: “Mental States,” “Brain Data”

The term “mental state” is used here to denote the diverse class of mental phenomena already familiar in day‐to‐day social and legal settings. These phenomena include sensory perceptions or experiences, emotional states, states of awareness or attention, desires, memories, and thoughts. This rough definition is a pragmatic one that is “folk psychological” rather than philosophical; in other words, it reflects the everyday way that human beings understand mental states and their relationship to intentional actions.^7^ This means that the discussion here sets aside the extensive philosophical debate regarding the nature of mental states and the meaning of the term. It is nonetheless true that uncertainty about the metaphysics of mental states generates important and deep questions. For example, the whole project of trying to identify a mental state and then to make social use of that information is on unstable ground if we are not sure what, if anything, a mental state is. Similarly, the debates over cognitive or mental ontology—the identification and classification of the types of mental states that do exist—will be discussed below in relation to efforts to correlate neural activity with mental states, but they will not be exhaustively discussed here.

This way of talking about mental states is justified here given that international organizations and multiple legislatures are already making legal policy to address the privacy of brain data in order to protect mental privacy.^8^ For example, a proposed bill in Minnesota would declare a “right to mental data” and goes on to restrict the collection of data about brain activity without consent in order to protect the right to mental privacy.^9^ Legislatures and courts are considering the legality of inferring mental states from brain data within the criminal justice system.^10^

One way to ascertain if a person is experiencing a mental state is to ask them, but there are many reasons that self‐report may be impossible, difficult, or unreliable. People may be unable to communicate. They may lack good introspective awareness of a particular mental state that may nonetheless be important to know about (for example, drowsiness may elude the drowsy person but be vital for determining capacity to drive or responsibility for an accident). Outright deception might be motivated by fear of social stigma or legal sanction. Or a person may communicate truthfully about their experience of a mental state yet simply be mistaken (due to a hallucination or a confabulated memory, for example).

Mental states can offer evidence about past or present events or permit predictions about future actions or states. Memories are routinely used to try to determine what a person has done or experienced, despite problems with perceptual errors, false memories, and occasional incentives to be untruthful. Mental states such as alertness or attention are relevant to medical evaluation and prognosis in brain injury, to student learning, or to performance in safety‐critical situations like driving and some employment contexts. Whether a person is in pain is important for medical purposes and also for evaluating legal claims for personal injury compensation. Brain‐computer interfaces (BCIs) can detect imagined acts—identifying imagined speech or movement, for example—making BCIs useful as assistive devices for people with communication or mobility impairments. In some jurisdictions, brain‐activity data is used to determine whether a person is concealing information or giving deceptive responses.^11^

Mental states may also be used prognostically to predict future events or behavior. For example, mental states such as those related to sexual interests are currently evaluated in forensic psychiatry for therapeutic purposes as well as in forensic risk assessment.^12^ Techniques are being developed to detect suicidal mental states in order to intervene in the care of people at risk of suicide.^13^

As noted above, “brain data” is used here to refer to information about brain structure or activity that could support a chain of inferences leading to a conclusion about a person's mental state. Sometimes, variation in brain structure can predict mental states, as suggested by research linking specific brain volume changes to the severity of phantom limb pain.^14^ In other cases, the brain data pertains not to structure but to activity, which is detected using techniques that measure changes in blood flow, electromagnetic fields, or markers of metabolic activity in the brain.^15^ These methods of detecting activity differ in both spatial and temporal resolution. For example, encephalography detects brain activity more directly than do techniques measuring blood flow or metabolism via radio‐labeled glucose uptake, but it has poorer spatial resolution.^16^ Each of these general approaches may be performed noninvasively, although electrical activity in the brain may also be collected with greater resolution using implanted electrodes. Among these techniques, functional magnetic resonance imaging (fMRI) has become very popular for research because it can capture whole‐brain data and does not expose participants to radiation.^17^ However, it is expensive and impractical for widespread day‐to‐day applications. These technologies are evolving, with portable devices to measure hemodynamic activity using functional near‐infrared spectroscopy^18^ and electromagnetic activity using refinements of magnetoencephalography.^19^

This data about brain structure or neurophysiological changes in hemodynamic, electrical, or metabolic variables must in turn be linked to the mental states one wants to learn about. This is done using sophisticated statistical and computational analyses to identify patterns across large datasets that correlate with particular mental states. The validity of the inferences from brain data to mental states, and the ethics of using those inferences in social contexts, will vary from case to case. Among the questions that need to be explored are these: What are the ethics of using inferences that are of uncertain validity? Do the ethics differ for predicting future behavior? Is it fair to treat an inference based on brain data as more authoritative than a person's self‐report about their experiences? Is there ever a right to have one's mental states inferred from brain data? And what are the contours of a right to mental privacy in an age of inferring mental states from brain data—how should we balance that right against the rights and interests of others?

It is worth noting that many of the regulatory and legislative discussions of neurorights and brain privacy use the term “brain data” without defining it, and those that do define “brain data” offer definitions that can be broader (including information about brain structure and activity^20^) or narrower (restricted to information about brain activity).^21^ As noted, this article will use “brain data” to refer to data about brain structure or activity that is being proposed or used as the foundation for the chain of inferences from the brain to a particular mental state. It does not include every possible piece of information about the brain, such as structural abnormalities in blood vessels, although this does not mean that the excluded data cannot be sensitive. Indeed, ethicists debate how to handle incidental findings about such abnormalities, which are sometimes detected in neuroimaging research.^22^

## The Inferential Chain from Brain Data to Mental State

**Figure 1:**
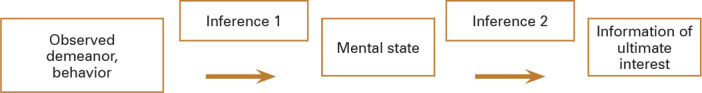
Traditional Inferential Chain

Human beings have long made guesses about other people's mental states and likely behavior using a chain of inferences from observed statements, acts, and demeanor. Sometimes, the mental state itself is of interest, such as when we want to know whether someone is in pain. Sometimes, the mental state is of interest because of what it says about another matter, such as when we are trying to predict future criminality or evaluate the veracity of a statement. In making these guesses, we rely on a chain of inferences from observations, and the reasoning process is often implicit, rapid, and based on learned patterns and expectations within a given cultural context. The ability to make such inferences is a critical social skill, as is the ability to shield one's mental states from others. A schematic describing this inferential chain is in figure 1. Various “high‐tech” approaches have been developed or attempted to infer mental states from observable physiological variables. Some examples are the polygraph,^23^ eye‐gaze tracking,^24^ and biomarkers of pain.^25^

Note that both inferences outlined in figure 1 rely on the use of patterns learned through prior observations, and as a result, the accuracy of the conclusions drawn depends on the validity of those learned patterns. For example, the ability to perceive and interpret changes in facial expressions develops early in life, but speed and accuracy continue to develop from experience into adulthood.^26^ A pattern that is learned from interactions with multiple people over time is a composite pattern that is often, but not always, accurate for assessing a given individual. This is referred to as the “g2i” (“group‐to‐individual”) problem in the neuroscientific context, where a statistical correlation between brain data and a mental state is established based on group data and that correlation is then applied to an individual.

Similarly, patterns learned in the course of social and emotional development may be nonspecific; a particular demeanor may be attributable to multiple mental states.^27^ For example, since human beings frequently smile when amused but may also smile in stressful and unpleasant situations,^28^ it would be a mistake to assume that a smile definitively establishes that a person is amused. This is an example of the “reverse‐inference” problem, often discussed in relation to cognitive neuroscience studies that establish a correlation between a mental state and a pattern of brain activity but then make the risky inference that the mental state exists whenever that pattern of brain activity is detected. If the neural pattern can occur with various mental states, then the reverse inference is shaky. In the context of human social development, an individual amasses a very large set of social interactions over a lifetime and becomes more knowledgeable about the range of mental states that could be associated with observable markers, as well as with the subtle distinctions between those markers. This rich information helps to reduce the riskiness of the reverse inference.

**Figure 2:**
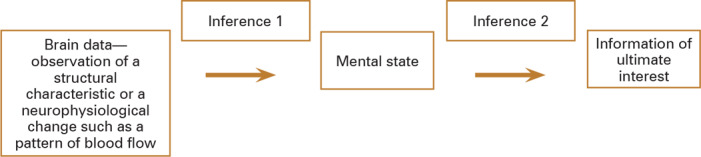
Inferential Chain from Brain Data

The use of brain data follows a similar inferential chain, represented by the simplified generic schematic in figure 2. As above, sometimes the mental state is the information of interest, while sometimes there are further inferences from that mental state to the matter of ultimate interest—an event in the past, a prediction of future behavior, veracity.

What is new about this second inferential chain is the use of brain data, generated through tremendously complex processes, including extensive data processing and statistical analysis, and reliant on implicit hypotheses about the brain. To gain a sense of the technical complexity, consider the relationship between blood flow and neuronal activity. The temporal and spatial increase in blood flow in response to increased neuronal activity is known as “neurovascular coupling,” which appears to allow an inference about neuronal activity from observations about blood flow. However, the relationship between blood flow and neuronal activity turns out to be a highly complex one, and increased blood flow can sometimes reflect something other than increased neuronal activity.^29^ BOLD fMRI (blood oxygen level‐dependent functional magnetic resonance imaging) relies on a further effect of the increased blood flow—namely, a reduction in the level of deoxyhemoglobin as an increased flow of oxygenated blood arrives.^30^ Multiple steps of data processing are required both to identify these changes and to generate an image of that activity.

Just as with traditional inferences about mental states, the reverse inference and the g2i problems are key issues for inferences based on brain data.

Research into the neural correlates of mental states often proceeds by selecting a mental state of interest, developing a method to bring about that mental state in experimental subjects, and then observing the brains of those subjects to see how their brains are different when they are in that mental state.^31^ This experimental structure yields information about how mental states are “encoded” in the brain, and it consists of a “forward inference” from the mental state to brain activity.^32^ “Decoding” is the reverse inference: it uses the information obtained experimentally but proceeds in the other direction, starting with an observation of brain activity and inferring the existence of the corresponding mental state. This inference works if there is a one‐to‐one correspondence between a given pattern of brain activity and a particular mental state. However, if the same pattern of brain activity could be associated with multiple states, then the inference is risky.^33^ For example, a study might reveal a pattern of brain activity when subjects report anxiety. A reverse inference would then observe that pattern and conclude that a person is anxious. But this inference is valid only if that pattern is specific to anxiety; if the pattern is consistent with other mental states, then the inference is invalid. Daniel Weiskopf points out that some regions of the brain are involved in “a wildly heterogeneous‐seeming array of activities across many domains,”^34^ making the reverse inference a substantial problem.

A further problem flows from experimental structure. The search for correlations between mental activity and brain data depends upon the specification of a target mental state to be studied and a method for reliably evoking that mental state.^35^ Both of these requirements are not easy to accomplish. There is great debate over the correct cognitive ontology—or, to put it another way, “the parts of the mind.”^36^ Even if a mental phenomenon of interest in the real world is specified correctly for study, it may be hard to evoke it in the laboratory in the same way as it would occur outside the laboratory. This poses the issue of ecological validity, or whether the research paradigm usefully reflects the real‐world phenomenon of interest. The problems are particularly acute with complex mental phenomena that must be simplified or reduced to subcomponents in order to be studied experimentally. For example, is the pattern of brain activity associated with falsely recollecting a word in a laboratory paradigm that is designed to induce false memories useful for identifying false eyewitness memories?^37^ Is a person's motivation to deceive in the laboratory the same as in a real interrogation in a courtroom setting?^38^ Some problems of ecological validity may be alleviated with methodological changes, such as the use of mobile devices to collect brain‐activity data in more natural real‐world settings^39^ or the use of virtual reality to mimic more naturalistic settings.^40^

Returning to the reverse‐inference problem, one influential school of thought regards it as a problem of probability that can be gradually mitigated through the acquisition of richer brain data about a larger number of mental states.^41^ In recent years, efforts have been made to do this through large‐scale meta‐analyses across many fMRI studies, relying on annotation of the studies to describe the corresponding mental states.^42^ Another approach is to use multiple forms of correlational evidence simultaneously, reducing uncertainty about the relationship between mental state and brain data by increasing the available information about the mental state. For example, Chuanjun Zhuo and colleagues have discussed an approach to diagnosing schizophrenia using a combination of multiple forms of structural and functional brain imaging along with other biomarkers, such as electrophysiological measurements.^43^

Machine‐learning techniques have been successfully used to make headway on more constrained problems—that is, rather than trying to identify which of a large set of highly heterogeneous mental states might be present, the task is to distinguish between variants of one mental phenomenon (such as which of several classes of objects or words a subject is perceiving).^44^ For example, using a large dataset of brain data associated with diverse visual phenomena, Shinji Nishimoto and colleagues were able to use machine learning to develop decoders that could reconstruct both static and dynamic visual experience from brain data.^45^ Brain‐computer interfaces rely on decoders trained to infer intended movements of the body to control prostheses^46^ or movements of the vocal tract to infer intended speech.^47^ Research to identify patterns of neural activity associated with semantic content may one day offer another form of communication neurotechnology.^48^ The development of accurate decoders is easier when they are trained for use by single subjects, but some decoders have been developed that work based on groups of subjects.^49^

The g2i, or group‐to‐individual, problem is a general and pervasive issue that applies whenever group data is used to make decisions about individuals. It also arises in evidence‐based medicine and forensic risk assessment in law.^50^ For inferences from brain data to mental states, the problem is whether a pattern identified in a group of research participants translates to a specific individual case. Many studies are based on observations of a group of research participants, and a pattern observed in those studies may not fit an individual outlier particularly well. In addition, any biases in the selection of the group can limit the ability to generalize to non‐group members if there are systematic group differences in brain activity associated with a particular mental state (for example, due to age‐related changes in the brain). By contrast, if a decoder is trained on and used on a single individual, then the g2i problem does not arise. (Similarly, for traditional “mind reading” based on normal social interaction, repeat experience with one person allows for a personalized pattern to be identified, as with studying an opponent to identify their particular bluffing “tells.”)

Many of the steps involved in inferring mental states from brain‐activity data have analogs in our traditional means of discerning the mental states and predicting the behavior of others. That said, the new brain‐based techniques include new inferential steps that rely, at least for the time being, on a relatively slim evidentiary foundation. It is necessary therefore to be quite cautious and to always keep firmly in mind the limitations of the inferences to be drawn. Neither a completely trusting nor a completely dismissive approach is likely to be desirable.^51^ The limitations of the inferential chain should be borne in mind in each particular case in which it is proposed that brain‐activity data be used to shed light on mental states or future behavior, and this method should be compared to the strengths and weaknesses of the default non‐brain‐based method of doing so, as well as to the implications of not drawing that inference at all using any method.

## The Ethics of Inferences from Brain Data to Mental State

A multitude of ethical issues arise when mental states are inferred from brain data. Some of these issues can be illuminated by existing applications of neurotechnologies, such as the use of electroencephalography‐based lie detection (also known as “brain fingerprinting”) in the criminal justice system in India. In other cases, a more speculative approach is necessary, using hypothetical developments to explore ethical issues that might arise should brain‐based methods be developed and put to use. It is important to be clear, however, about where the discussion is more speculative, given the importance of avoiding undue hype in neuroethical discussions.^52^

Whether and how ethical issues emerge depend on many factors, including the properties of the technologies themselves, such as how well they perform (or are believed to perform), how easily they can be used, and whether they can be used surreptitiously.^53^ Some commentators try to shelve ethical concerns about techniques for accessing brain data on the grounds that they cannot be used without the subject's cooperation, but this is a mistake. Some people have a medical need to use a neurotechnology that collects brain‐activity data, such as closed‐loop deep‐brain stimulation. They will have reason to cooperate, and they deserve protection from any risks associated with use of their brain data. And even if they lack such reasons to cooperate, it is relatively easy to induce people to cooperate by offering a benefit (like a discount on insurance fees or access to a wellness or gaming service) or by conditioning access to employment on cooperation. This is a well‐understood weakness of consent‐based privacy‐protection regimes. Similarly, it is clear that, in some contexts, people will ask to have their minds “read” if assumptions are made about them that they wish to dispel. Relying on the need for cooperation or informed consent as the main protection of the individual and answer to ethical concerns is inadequate as a blanket response.

One important issue is the standard for reliability of these inferences. At first glance, a very high level of reliability would seem to be a sine qua non for any reasonably high‐stakes application of these methods of inferring mental states from brain data. Yet there is a live question here, since the reliability of a novel method of gaining information about a person must be evaluated relative to the reliability of existing practices and alternatives.

Another set of ethical questions arises over trying to extend the inferential chain forward in time—predicting future mental states. For example, what are the ethical constraints on using brain data in forensic risk assessment in the criminal justice context? Here, the question of the validity of the inferential chain is sometimes extended by an additional inference from a current mental state to a future event or behavior. Beyond validity, self‐fulfilling prophecies, or the reinforcing social and psychological effects of these predictions, must be considered.

A third question has to do with the ethics of disregarding a person's subjective account of their mental state (“I remember x”) in favor of an account revealed by their brain‐activity data, which shows a false memory. Beyond the issue of whether and when it is fair to do this, there are other interesting and subtle ethical questions. For example, psychologically harmful self‐doubt might be instilled if a person defers to interpretations of their own brain data and comes to question their own perceptions.

Often, discussions of drawing inferences about mental states from brain data center on harms to privacy and freedom, but a fourth question to ponder is whether there should sometimes be a right to have such an inference drawn. Perhaps, for example, there is an obligation to draw these inferences in circumstances where it is the only way to give voice to people who are unable to communicate effectively due to locked‐in syndrome.

The individual interests at stake may be in competition with interests of others; this is particularly the case with privacy, where complex trade‐offs must be made between individual interests and the countervailing interests of others. A fifth set of ethical questions, then, would be what, if any, limits to put on the collection, use, and disclosure of brain data to protect mental privacy, how to think about ownership and control of brain data, and how control over one's own brain data should be balanced against the individual and collective interests of others.

## The Reliability Problem

The question about the reliability threshold that should be required of a technique of inferring mental states from brain data is fundamental for understanding the inferential chain from brain data to mental state and for addressing all of the following questions listed above. It will be essential to know the potential error rate before using the technique in high‐stakes contexts. However, in a context where we already draw inferences about mental states, perhaps the question should be less about what threshold of reliability should be required and more about whether the brain‐based inference is better and should replace or supplement current methods. And, furthermore, is reliability the only value that is relevant to the threshold for using the technique?

It is obvious that a useless technique should not be used. That would be at least unhelpful and wasteful, and possibly quite harmful. If a flawed technique nonetheless offers better information about mental states than current methods do, then there is a prima facie reason to consider using the technique to replace or supplement existing methods. But even if a brain‐based method would improve existing methods, there may still be reasons not to use it. The method may be expensive, impractical, and inaccessible and therefore unjustified despite the improvements it might offer. Another reason to question the use of a brain‐based method is that it would install an opaque and technocratic system in lieu of a more comprehensible and familiar, albeit less reliable, system of guessing at other people's mental states.^54^ This evaluation should be made case‐by‐case, as the trade‐offs will depend upon the characteristics of the brain‐based method proposed, what it offers relative to other available methods, and what the default outcome will be if no information about a person's mental state is available.

To illustrate the trade‐offs, consider the current use of a so‐called brain‐fingerprinting or brain‐mapping technique within the criminal justice system in India. The example shows how even a relatively unproven technology can address certain social needs and end up being adopted even if the scientific foundation is still uncertain.

“Brain fingerprinting” refers to the use of electroencephalography to detect a characteristic neural response thought to be associated with the recognition of a significant stimulus.^55^ The idea is that the recognition of an unusual feature of a crime that should be known only to a perpetrator or witness will help to establish that a suspect was present at the scene. Brain‐fingerprinting evidence has been rejected as inadmissible by several American courts because it does not satisfy the criteria for the acceptance of novel scientific evidence.^56^

A similar approach, known as “brain mapping,”^57^ has been used in the Indian criminal justice system at least since 2002, when it was first mentioned in a published legal decision.^58^ Brain mapping is offered through various state forensic scientific services, which also offer techniques such as the polygraph and narcoanalysis (administration of hypnotic drugs like sodium pentothal or “truth serum” during interrogation).^59^

The Indian criminal justice system is unusual in using brain mapping, and it has been vigorously criticized by forensic science experts in India.^60^ Why, then, is it still used? A review of the published cases in which brain mapping is mentioned offers some answers to this question. Essentially, its appeal lies in part in the hope that it may offer an answer to long‐standing systemic problems with the policing and criminal justice systems in India. Policing in India is plagued with long‐standing concerns about police brutality, and the public tends to regard the police as corrupt and partial.^61^ The under‐resourcing of Indian policing contributes to a large backlog of criminal cases, and there is a risk of false accusation stemming from the pressure to resolve the backlog of open investigations. A public‐policy nongovernmental organization in India—Common Cause—surveyed nearly 16,000 people in twenty‐two states in 2017 and found that 44 percent expressed a lot or some fear of the police, including of being beaten or falsely implicated in a crime.^62^

It is against this backdrop that the appeal of brain mapping must be understood. In the 2010 case of *Selvi*,^63^ the Indian Supreme Court addressed the question of whether brain mapping could be administered involuntarily. In its judgment, the court commented upon the problem of police brutality, noting without endorsing the suggestion that “the promotion of these techniques could reduce the regrettably high incidence of ‘third degree methods’ that are being used by policemen all over the country.”^64^ The idea that the adoption of scientific interrogation methods might help avoid police brutality has also surfaced outside India; Jinee Lokaneeta documents how “truth serum” and the polygraph were historically floated as forms of “humane third‐degree” in the United States.^65^

Another apparent function served by brain mapping is to alleviate the burden of the large backlog in criminal cases and to enable falsely implicated people to extricate themselves from the system. Lokaneeta observes that “the Indian criminal justice system is notorious for its backlog of cases,” meaning that a large number of the accused who do not get bail may spend years awaiting trial.^66^ For example, in *Jaga Arjan Dangar v. State of Gujarat*, the accused maintained that he had been falsely accused of murder and sought release on bail. He argued that the investigation had been neither fair nor impartial and that he had no other way to establish his innocence than brain mapping and polygraph testing.^67^ Similarly, the accused in *Rajan v. State of Kerala* ^68^ denied accusations of sexual assault and volunteered to “prove his innocence by subjecting himself to any scientific investigations.”^69^ The court criticized the investigation for “callous indifference and negligence” that allowed the real culprit to escape and subjected the accused to three years of unnecessary incarceration.^70^

There has been some pushback against the use of brain‐mapping evidence. The prosecution in *Jaga Arjan Dangar* argued against granting the accused's request for brain mapping on the basis that allowing every accused person to demand these tests would “create havoc” and “derail the entire machinery.”^71^ Despite these concerns, some courts, such as the High Court of Gujarat, have been very sympathetic, noting the desirability of “scientifically conducted tests, performed by … qualified experts” in order not just to find the guilty but to eliminate innocent parties as suspects.^72^

There have been some suggestions from outside India that brain mapping could serve a useful purpose in criminal justice. John Danaher suggests that the test could help with the problem of plea bargaining, in which an innocent accused has a strong incentive to plead guilty to some lesser offence to avoid a trial and possibly a greater punishment if convicted.^73^ His argument is that the willingness to undergo a valid test signals innocence, which could improve the plea‐bargaining process. This would be a useful signal only if there were a risk that an adverse result could be used against the accused at trial. Canadian courts tend to discount offers to take a polygraph test on the basis that the accused risks nothing because the results are inadmissible.^74^ However, a case from India demonstrates Danaher's point about the signaling power of the willingness to undergo brain mapping. In *Sukhdeep Singh and Iqbal Singh v. CBI*,^75^ a judge observed that, if Singh were guilty, “he would not have the guts to throw a challenge to the top brass of the Police Department to subject him to [lie detection, brain mapping, and narcoanalysis tests].”^76^

It is easy to criticize a criminal justice system for adopting a relatively unproven technique like brain mapping, and many people within India are quite critical. No system should adopt a technique that is useless. However, in a context like the justice system, where some form of credibility assessment is inescapable, the question should be whether a new technique offers something better than the status quo.

The usual approach of evaluating a speaker's demeanor and the general plausibility of the speaker's assertions is so familiar and largely intuitive that it can hardly be called a “method.” It is also subject to a whole range of known flaws and biases, with certain speakers more apt to be believed or disbelieved on irrelevant grounds like physical attractiveness.^77^ The model jury instruction recommended by the Canadian Judicial Council directs judges to warn juries not to jump to conclusions based on demeanor.^78^

Assuming that brain mapping is a reasonable approach that is better or no worse on average than the usual methods (admittedly, a big assumption) and that it offers a means of exculpation to people who are apt to be disbelieved, there may still be reasons not to use it. The techniques may be expensive and impractical. In addition, past judicial encounters with expert scientific evidence reveal concerns about the “dehumanization” of the justice system,^79^ as well as the technocratic takeover of justice by experts whose evidence is “highly resistant to effective cross‐examination by counsel who are not experts.”^80^

An accused person may have a strong reason to argue that a fair right to make a full answer and defense must allow them to offer brain‐based evidence, and it is no accident that the U.S. cases dealing with brain fingerprinting all involved efforts by accused people to clear their names. It is not enough to dismiss these techniques out of hand. There are a range of competing values to be balanced, and the question is whether a brain‐based technique is an improvement over the status quo, and for whom.

## Understanding What We're Doing

Technologies capable of collecting detailed information about brain structure and function, in tandem with big‐data technologies that help interpret that data, raise ethical questions that will only grow more urgent. Invasiveness, expense, and impracticality or poor reliability will all independently hamper the widespread uptake of these technologies. However, considerable progress is being made in refining existing techniques for noninvasive collection of brain data^81^ and in developing new ones.^82^

Making inferences about the mental states of others based on their observed behavior is a quotidian aspect of human social life, and many of the problems associated with the inferential chain in that context recur with brain‐based inferences. It is also true that most or maybe all of the brain‐data techniques for inferring mental states remain experimental and unproven for broad use in social decision‐making. They should be approached with caution.

That being said, it is important to avoid status‐quo bias. The current methods for making judgments about the mental states of others also have flaws, and the risks of these are not evenly distributed. Some people are more likely than others to be disbelieved in courts, for example, and their claims of pain are more likely to be dismissed. Of course, resolving social biases by developing opaque and expensive technologies for studying brains is clearly inferior to addressing those biases directly. But while we work on that front, techniques that offer incremental improvements in accuracy beyond the status quo—and at reasonable cost—are difficult to refuse.

In anticipation of this pressure, it is crucial to pursue research that can refine techniques for collecting and interpreting brain data and also to conduct research that measures the reliability of inferences from brain data to mental state. If we do not understand how well the inferential chain works, we might misuse the technologies. They could offer a seductive but illusory solution to a social problem. Conversely, we might underuse them, failing to recognize that they offer real improvements over familiar but flawed and inadequate ways of understanding mental states.

## Acknowledgment

This article is part of a series, Neuroscience and Society, whose development by The Hastings Center is funded by the Dana Foundation.

This work was conducted within the HYBRIDMIND project, funded by the ERANET‐Neuron Program and the Canadian Institutes of Health Research.

## Disclosure

The author is a member of an external advisory board for INBRAIN Neuroelectronics.
